# Heterogeneous network approaches to protein pathway prediction

**DOI:** 10.1016/j.csbj.2024.06.022

**Published:** 2024-06-27

**Authors:** Gowri Nayar, Russ B. Altman

**Affiliations:** a Department of Biomedical Data Science, Stanford University, United States; b Department of Genetics, Stanford University, United States; c Department of Medicine, Stanford University, United States; d Department of Bioengineering, Stanford University, United States

**Keywords:** Protein pathways, Heterogeneous networks, Embeddings, Functional proteomics, Pathway prediction

## Abstract

Understanding protein-protein interactions (PPIs) and the pathways they comprise is essential for comprehending cellular functions and their links to specific phenotypes. Despite the prevalence of molecular data generated by high-throughput sequencing technologies, a significant gap remains in translating this data into functional information regarding the series of interactions that underlie phenotypic differences. In this review, we present an in-depth analysis of heterogeneous network methodologies for modeling protein pathways, highlighting the critical role of integrating multifaceted biological data. It outlines the process of constructing these networks, from data representation to machine learning-driven predictions and evaluations. The work underscores the potential of heterogeneous networks in capturing the complexity of proteomic interactions, thereby offering enhanced accuracy in pathway prediction. This approach not only deepens our understanding of cellular processes but also opens up new possibilities in disease treatment and drug discovery by leveraging the predictive power of comprehensive proteomic data analysis.

## Graphical Abstract

**Figure:**
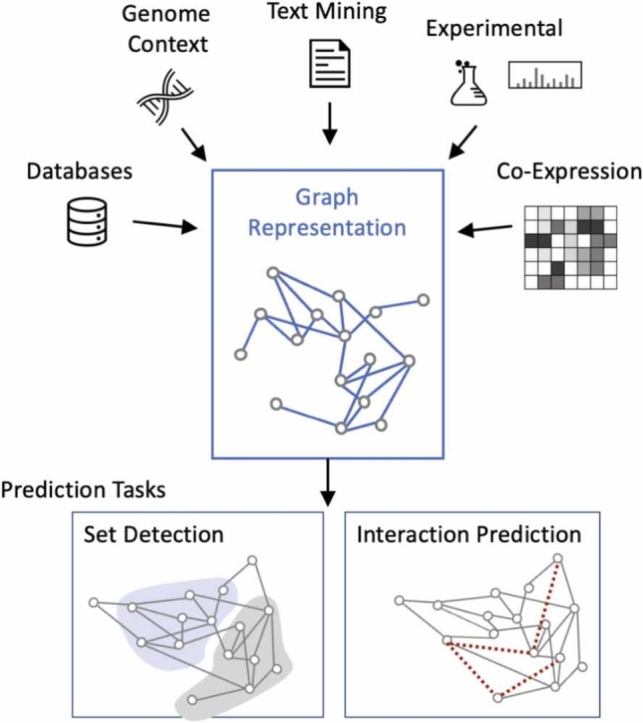
Understanding of protein pathways are crucial to understand human cellular responses and disease responses. Five categories of data sources are used to build the interaction network. The types of interactions are those (1) stored in publicly available databases, (2) inferred from the genome, (3) inferred from published literature, (4) found through physical experiments, and (5) inferred from expression studies. Network-based methods have emerged as the most effective abstraction to model protein-protein interactions (PPIs) and predict protein pathways, and so the interaction types are combined into one network. Two pathway prediction tasks use this network as input - (1) predicting the subset of protein nodes involved in a pathway and (2) predicting the interactions within a pathway. In this review, we describe common PPI data sources, and discuss the implications of each data type on the network model.

## 1 Introduction

The failure to diagnose and treat diseases often stems from a lack of understanding of the mechanisms of action and associated protein pathways. A pathway is a series of interactions among proteins in a cell that causes either the assembly of new molecules, changes in gene regulation, or changes in cell state. Pathways combine the function of individual proteins to create complex cellular responses. Most cellular tasks do not follow a linear sequence of protein-protein interactions (PPI), but instead create a branching, interconnected set of interactions. Pathways exist within a network of complex interactions and so represent a useful subset of all possible PPIs across the proteome. Distinct boundaries between pathways are a useful simplification because they can be associated with specific phenotypes [1]. Pathway representations provide a summary of (1) the proteins that compose the pathway, (2) the individual interactions between the proteins, and (3) the overall functional and phenotypic significance of the proteins and their interactions.

There is a gap between current data analysis methods and their ability to derive precise functional information from molecular data. Obtaining lists of genes/proteins that are differentially expressed between two phenotypes is common, but determining the series of interactions that cause these changes remains challenging. The individual protein-protein interactions (PPI) within the pathway are discovered experimentally through studies of cultured cells, bacteria, yeast, and other model organisms, each with varying biases and degrees of confidence. PPI experimental methods include in vitro techniques such as co-immunoprecipitation, pull-down assays, yeast two-hybrid assays, and protein microarrays, and in vivo methods like fluorescence resonance energy transfer (FRET), bimolecular fluorescence complementation (BiFC), and reporter gene assays [2], [3], [4]. Modern high-throughput sequencing technologies generate different data types, including genomic sequences, gene expression profiles, and combined transcriptomic and protein expression information; this data does not directly provide pairwise protein interactions, so post-processing methods are required to deduce the relevant interactions. Inferring the pathways responsible for a phenotype from the full set of proteomic interactions is essential. Therefore, in this review we focus on pathway analysis methods that learn from known interactions to generate novel predictions.

**Fig. 1:**
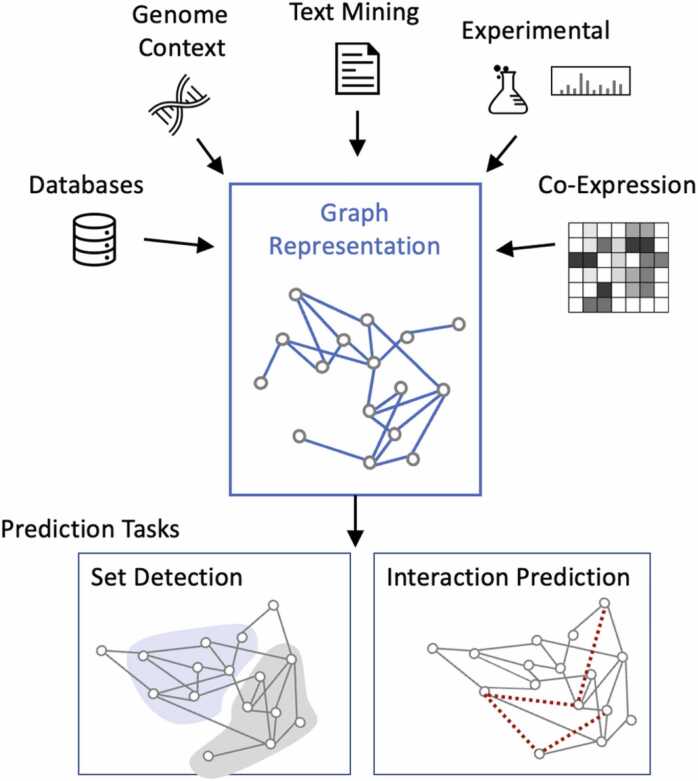
Understanding of protein pathways are crucial to understand human cellular responses and disease responses. Five categories of data sources are used to build the interaction network. The types of interactions are those (1) stored in publicly available databases, (2) inferred from the genome, (3) inferred from published literature, (4) found through physical experiments, and (5) inferred from expression studies. Network‐based methods have emerged as the most effective abstraction to model protein‐protein interactions (PPIs) and predict protein pathways, and so the interaction types are combined into one network. Two pathway prediction tasks use this network as input - (1) predicting the subset of protein nodes involved in a pathway and (2) predicting the interactions within a pathway. In this review, we describe common PPI data sources, and discuss the implications of each data type on the network model.

Manually curated pathways are annotated from interaction literature and have a high true positive rate of pathway inclusion. These are regarded as gold-standard pathways, available in public databases such as Reactome Pathway Knowledgebase, Kyoto Encyclopedia of Genes and Genomes (KEGG), MetaCyc Metabolic Pathway Database, and Gene Ontology (GO) [5], [6], [7], [8]. However, these pathways often miss interactions and proteins that are discussed infrequently in the literature, causing a high false negative rate. Instead, we focus on computational methods that perform across the entire proteome, including proteins and interactions with varying amounts of experimental data. Network biology is a common tool for modeling complex biological phenomena for computation at scale. In order to leverage modern machine learning methods, we model proteomic interactions as a network, where the nodes represent proteins and an edge represents an interaction between two proteins. Heterogeneous networks, a subtype of network that can distinguish between different node and edge types, are useful for representing the different data sources for interaction and the range of protein annotation [9]. Thus, in this review, we focus specifically on heterogeneous network methods for pathway analysis. We review the approaches to (1) representing the different interaction data sources within the network, (2) pathway prediction, and (3) pathway evaluation. Fig. 1 shows the flow of generating protein interaction data, building a network, and the downstream pathway prediction tasks. Pathway prediction tasks involve identifying relevant proteins and predicting pairwise interactions that form the pathway. In heterogeneous networks, edges (interactions) can be directional and labeled to describe the interaction type, which is crucial for pathway representation. Evaluation ensures biological applicability for future tasks, such as mechanistic explanations of disease or drug target development. .

**Table 1:** Commonly used, publicly available pathway databases and their size of human data. These databases contain high-quality pathways that have been manually curated, and thus are used as gold-standard examples for prediction algorithms. However, manual creation misses many pathways, explaining the limited number of human proteins present in each database. The Gene Ontology database does not have direct pathway data, but it does provide biological process annotation for each protein and thus, similar functional proteins that share a biological process are often associated together within a functional group.

Database	Description	Human Proteins	Human Pathways
Reactome	A manually curated database that provides detailed information on human biological pathways and processes, enabling the discovery of functional relationships in gene expression and disease data.	11,442	2698
KEGG Pathway	Maps molecular interaction, reaction, and relation networks for metabolism, genetic information processing, environmental information processing, cellular processes, and human diseases	21,509	257
MetaCyc	Curated database of experimentally elucidated metabolic pathways and enzymes from all domains of life, including humans, supporting metabolic engineering and comparative genomics	2709	135
Gene Ontology	Resource initiative provides a standardized vocabulary to describe gene and protein functions across species, covering molecular functions, biological processes, and cellular components	20,299	Not directly applicable

### 1.1 Network Fundamentals

This section describes the fundamental network concepts used by the following pathway prediction algorithms for heterogeneous networks. We provide a brief overview of the relevant topics and vocabulary. Xia *et al.* and Grindrod *et al.* provide reviews focused on graph theory and network concepts in proteomics [10], [11]. For a focus on the prediction algorithms, Zhou *et al.* provides a comprehensive review of graph neural networks and Zhang *et al.* reviews graph convolutional networks [12], [13].

A basic network consists of a set of *nodes* and a set of *edges* that define relationships between the nodes. In the PPI network model, the proteins are represented by nodes and their interactions are represented by edges. With a *heterogeneous network* model, we can add unique *attributes* to the protein nodes (structure representation, sequence representation, tissue expression) and to the edges (confidence of interaction, experimental source) [14]. These attributes are vectorized, assigning distinct numeric values or vectors to each feature and aggregating them to create a comprehensive vector definition, thus incorporating multiple data types into a single representation for prediction methods. Thus, this representation incorporates several data types into one representation that is passed on to the prediction method. A node’s *degree* is the count of edges that stem from that node, and so in the PPI network model, it represents the number of interactions for a protein. PPI networks are *scale-free networks*, which means that the distribution of degrees follows a power law [15], [16]. Thus, a vast majority of the protein nodes have very few connections, while a few nodes have a high degree. These high-degree nodes are called *hub nodes* and are typically the well-studied proteins that are published often in the literature [17]. Hub nodes are often over represented in predictions; since they have the highest number of edges, they appear most frequently in the training set, and thus the model will tend to make predictions for hub nodes more often than low-degree proteins [18]. The *clustering coefficient* measures how connected a node's neighbors are to one another, quantifying the abundance of connected triangles. A PPI network has a high clustering coefficient thus following *small-world* principles [19]; there is a high probability of a short path between any two protein nodes. Typically, these paths will traverse through a hub node, demonstrating the importance of the hub nodes in the network topology. Small-world networks are also more likely to contain subgraphs of *cliques*, where all pairs of nodes in the subgraph share an edge [20]. In the case of PPI networks, these cliques are often considered to be evidence for functional pathways. However, the human PPI network is incomplete, with an estimated 80 % of missing edges [21], and thus, relying on cliques to identify pathways has a high false negative rate. The *matching index* measures the similarity between two nodes in a network by comparing their shared neighbors. This measure identifies functionally similar proteins without requiring that they share an interaction edge.

**Fig. 2:**
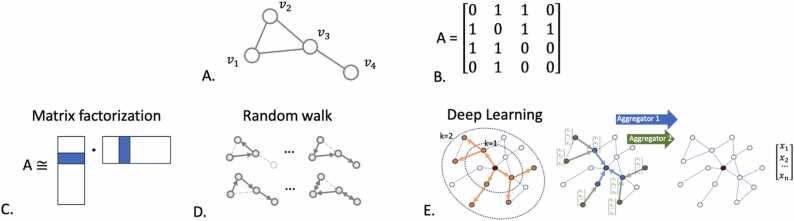
Example of a graph and the three types of embedding methods. A provides the example graph with 4 nodes. B represents the adjacency matrix that defines the example graph. Each row *i* and column *i* corresponds to node v*i.* When the position (*i, j)* has a one in the matrix, this denotes that there is an edge between node *i* and *j.* C shows how matrix factorization can be used to define a vector for each node. D shows the creation of random walks through the graph. This set of sequences of nodes is used as input to a neural network that defines a vector for each node. This resulting vector for node *i* is such that the value at position *j* is equivalent to the probability of transitioning between nodes *i* and *j.* E shows how deep learning methods use the neighborhood of the red node and aggregate their vectors to update the definition of the red node. Each deep learning model uses a different aggregator, which could include the mean, max, or a nonlinear function. The nodes at further distances in the neighborhood typically have less weight in the aggregation.

A network can be stored as an *adjacency matrix* or a *sparse matrix*. An adjacency matrix is an *nxn* matrix, where *n* is the number of nodes in the network, and the value at matrix [*i, j*] represents the presence or absence of an edge connecting node *i* and *j.* For heterogeneous graphs, this value is replaced by the attributes that describe this edge [22]. In a sparse matrix implementation, only non-zero values are stored, so two nodes that do not share an edge are not maintained in the matrix [23]. Modern machine learning methodologies use vectorized representations of the network, called *embeddings*, to transform the high-dimensional data into a lower-dimensional space while preserving the properties of the original network [24]. An embedding is a representation of the node in vector space, where each node is assigned a vector that captures its relational and structural characteristics within the network. For each protein node, the vector length is typically on the order of 1000 [25]. These embeddings are created using techniques such as *matrix factorization*, *random walk* methods, and *deep-learning* approaches. Matrix factorization methods decompose the adjacency matrix. A random walk is generated by performing a series of one-hop transitions across the network; the probability of traversing an edge can be a function of the attributes of that edge, typically the confidence score. Random walk methods, such as node2vec [26], simulate random pathways through the network and use the sequences of nodes as input to a neural network to learn the vector representation for each node. Deep learning approaches, such as *Graph Convolutional Networks* (GCN) or *Graph Attention Networks* (GAT) [27], [28], use layers of convolution and attention to aggregate the features of a node’s neighbors and perform an update function to the node’s vector. Attention mechanisms in neural networks focus on parts of the input data most relevant to the task at hand, improving the ability to capture relationships within the data. These methods enable the extraction of latent features, correlations, and mutual information from PPI networks, which are otherwise too complex to analyze directly. The embedding methods are described graphically in Fig. 2.

We review algorithms that predict pathways, or functional subgroups, from the larger PPI network. Most of these methods use deep-learning approaches to perform this prediction, specifically *Graph Neural Networks* (GNN). There are several types of GNN, but in this review, we focus on the most common methods - GCNs and GATs. A GCN is similar to a traditional Convolutional Neural Network, as it learns features or embeddings for a node by convolving over its neighbors. These embeddings are then passed to dense layers that use non-linear activation functions to determine if the input passes a predefined threshold, thus generating predictions. GraphSAGE is an example of a GCN that has gained prominence in pathway prediction tasks as it handles heterogeneous graphs, aggregating features from neighboring nodes differently for each edge type traversed [29]. The key difference between a GCN and a GAT is how the information from the neighboring nodes is aggregated. The GAT introduces attention over the features of the neighbors in the convolution operation, instead of a simple mean aggregation. The GAT has the ability to learn alternative network edge weights, allowing it to downweight or upweight edges based on their importance for the network reconstruction task. These types of networks are generalizable to several tasks, including (1) *node classification*, (2) *link prediction*, (3) *community detection*, and (4) *graph classification*. These four tasks encompass the tasks performed by the pathway prediction algorithms reviewed: (1) Node classification predicts a missing label (functional annotation) for a node, (2) link prediction predicts an edge (an interaction) between a pair of nodes, (3) community detection divides nodes into distinct subgraphs (pathways), and (4) graph classification predicts a category (phenotype) for a subgraph (pathway).

A recent advancement in the field has been driven by the use of transformer models. Transformers leverage a self-attention mechanism, which enables them to consider the relevance of each token (protein node) in a sequence to every other token, effectively capturing complex dependencies and relationships across the entire input data [30]. This mechanism operates in parallel, allowing transformers to process data more efficiently and handle longer context windows than their predecessors. Bidirectional Encoder Representation from Transformers (BERT) is a commonly used transformer model which can capture training context from tokens in both directions within a sequence, improving the contextual understanding [31]. This type of architecture importantly generates sequential predictions, which is crucial to predicting the cause-and-effect nature of protein pathways. While the application to PPI networks is still nascent, these methods show promise in enabling the discovery of new pathways.

## 2 Pathways are built from diverse evidence for individual protein interactions

In this section, we review methods for constructing the interaction network and their implications on downstream pathway prediction. Individual protein interactions are building blocks of pathways. To facilitate proteome level analysis, experimental evidence from the literature is collected into publicly available primary databases (BioGRID, HIPPIE, BIND, CORUM) [32], [33], [34], [35]. Experimental interaction data has a high true positive rate but is limited in the number of proteins included and thus can suffer a high false negative rate. To increase the number of proteins and interactions included, secondary databases (STRING DB, hPrint, APID) store both experimental data (obtained from existing primary databases) and derived interactions from genome contexts and gene expression analyses [36], [37], [38]. This combination helps to address the issue of sparsity in the PPI network. However, integrating several data sources risks abstracting away the biological nuances that qualify each interaction (cell type, spatial and temporal context, environmental factors). Heterogeneous network representations allow each edge to be labeled with these metadata. Therefore, secondary databases that denote the distinctions between each edge type are more suitable as input for pathway analyses.

Many methods for pathway analysis use STRING DB as input, the largest secondary database with the highest number of human PPIs [18]. STRING DB maintains 5 types of interaction data - genome context, co-expression, experimental, databases, and text mining. Genome context predictions are inferred from the spatial relationship of the genes/proteins on the genome under the assumption that if two genes/proteins are consistently located close together, they are functionally linked. Co-expression interactions follow a similar type of logic, where these interactions are inferred by detecting genes that are consistently expressed together across various conditions and tissues. Experimental interactions are identified through high-throughput methods, such as yeast two-hybrid, screening, and mass spectrometry. The database interactions include interactions that are already documented in the primary databases; STRING specifically includes interactions from BioGRID, DIP, IntAct, MINT, and HPRD. Interactions from text mining use natural language processing techniques to identify and extract mentions of potential interactions from PubMed literature. This type of interaction is the noisiest, as these interactions are only hypothesized or implied in the text. For each of these categories, STRING reports an association score that reflects the likelihood of the interaction being biologically accurate. Most pathway prediction algorithms begin with an interaction filtration step; it is common to omit the low-confidence categories, such as genome context and text mining [15]. The association scores from the remaining three categories are summed, and interactions that do not meet a selected threshold are excluded [15]. This increases the likelihood that the downstream pathway analyses learn from the broadest set of interactions that meet the necessary quality requirements.

The most common interaction types require care consideration, in particular physical interactions identified through experimental analysis and interactions inferred from co-expression studies. While the database interaction category from STRING is also often used, it fundamentally originates from experimental analyses, so we consider these two interaction types similarly. We summarize methods to computationally represent experimental and co-expression interactions to account for their biological significance and biases.

### 2.1 Experimental interactions

Interactions from experimental data are highly likely to be biologically relevant because they provide direct evidence of interactions between proteins. They also provide important metadata about conditions under which the interaction occurs, which helps describe the biological context for the relevant pathway. Therefore, these are the most commonly included edge types for pathways analysis.

The specific physics of in vitro and in vivo experiments dictate which PPI are discovered. For example, co-immunoprecipitation (co-IP) is a common antibody-based experiment used to find individual interactions, but requires the existence of specific antibodies for the target proteins and is generally restricted to studying interactions among soluble proteins, leading to a potentially high false negative rate depending on the experimental conditions (e.g. ineffective antibodies, dynamic nature of interactions) [39], [40]. High-throughput experiments, such as yeast two-hybrid (Y2H) and tandem affinity purification mass-spectrometry (TAP-MS), experiments expand the set of studied proteins by capturing tens of thousands of interactions with one experiment [2]. These non-specific assays used in high-throughput experiments have a risk of false positives, which can be mitigated with multiple screens [41]. In addition to different proteins detected, the types of interactions detected vary with experiment type; Y2H methods produce direct pairwise physical interactions, while others denote an interaction when two proteins are present on a larger protein complex [2]. Thus, this heterogeneous data necessitates a representation method that supports storing the relevant attributes for each node and edge. By using a heterogeneous graph representation of the input PPI network, the edges can be annotated with the experiment type and conditions to qualify the interactions the pathway analyses build upon.

To adjust for each edge type in the pathway analysis, the impact of the experimental differences and the confidence in the resulting interactions must be quantified. STRING DB, along with the other secondary databases, provides an ad hoc association score. In STRING, the experimental association score corresponds to the probability of finding the linked proteins within the same KEGG pathway and accounts for the frequency or reciprocality of the detection. Y2H-SCORES is a statistical framework that calculates confidence scores for Y2H screens (fusing two proteins of interest to different parts of a yeast transcription factor, and if the proteins interact, the different yeast parts come together). Y2H-SCORES employs quantitative ranking scores to evaluate the interactions: an enrichment score to measure significant enrichment for each positive interaction and a specificity score to assess the specificity of the two target proteins interacting compared to interacting with other proteins[42]. The enrichment and specificity scores account for the experimental design (eg. one vs. many target proteins for each interaction), normalization (eg. transcripts per million), and controls (eg. non-target proteins), quantitatively describing the confidence in the PPIs resulting from the Y2H screens [42]. Velasquez-Zapata *et al.* showed that this weighting method facilitated the discovery and validation of novel interactions between the nucleotide-binding leucine-rich repeat (NLR) immune receptor MLA6 and fourteen proteins, including interactions required in signaling, transcriptional regulation, and intracellular trafficking [43].

The variance and noise in differing experimental conditions introduces erroneous interactions in the network model, and the errors propagate to downstream prediction tasks. Unfortunately, the overlap between the PPIs identified by different high-throughput studies remains low, with each method producing interactions unverified by others [44], [45]. Furthermore, contradictions often arise when extracting interactions from prior physical experiments in the literature [45], [46]. Dunham *et al.* [47] quantify the impact of noise on interaction network predictions; they find that most PPI inference methods use evaluation datasets that contain a higher proportion of positive interactions than found in the current known human PPI network. When these prediction algorithms are tested on noisy, contradictory datasets that are representative of the human PPI network, they do not achieve good performance. Instead, the predictions are over-influenced by the well-characterized proteins found in the literature and perform poorly in the data-poor regions of the proteome[47]. This noise must be considered when developing the pathway prediction model. While several methods (Section 4) use machine learning tools to address the noisy data, accounting for the noise and contradictions in the PPI network requires further development.

### 2.2 Interactions deduced from co-expression studies

Co-expression studies are considered a silver standard interaction source for PPI networks. RNA expression data provides context-specific data that can be used to detect co-occurring proteins given a particular phenotype [48]. Co-expression is a useful indicator of potential interaction as proteins that are involved in the same pathway are expressed together. Though the expression pattern is not direct evidence for the interaction, it is a useful proxy metric that also relates the identified interaction or pathway to the phenotype. Barker *et al.* [49] used RNA-seq expression of breast cancer cells to discover disease-indication-specific signatures. They constructed phenotype-specific networks to identify cell signaling subnetworks that were previously omitted from the pathway. This work showed that high-throughput analyses can avoid the bias towards well-studied proteins.

Gene expression-based methods, such as NanoString [50] and L1000 [51], perform high-throughput quantification of transcriptomic data to identify patterns that govern biological functions (response to drug, pathways in disease, regulatory network). The individual experimental expression data is stored in publicly available databases, such as the Gene Expression Omnibus (GEO) [52] and the Human Protein Atlas [53], aggregated for proteome-scale analysis. This data does not directly imply pairwise protein interactions, so post-processing methods are required to deduce the relevant interactions. A correlation metric is used to construct the PPI network from the expression data. However, expression data tends to be sparse, highly redundant, and high-dimensional when considering the full proteome. Therefore, using simple correlation metrics such as the Pearson Correlation Coefficient (PCC) has low performance. Instead deep learning methods are more often used to also capture non-linear relationships. A novel method, Functional Association using Variational Autoencoders (FAVA), has gained use in analyzing expression data. Variational Autoencoders (VAE) are a type of neural network that consists of an encoder and a decoder. The encoder compresses the data to a lower-dimensional space and the decoder reconstructs the data from the low-dimensional representation, thus learning a representation that maintains the key features of the input data. FAVA uses the VAE to reduce the dimension of the expression data and calculates the distance between the proteins in this low-dimensional space. Proteins that are close to the low-dimensional space are predicted to have functional associations. This method reduces the sparsity and redundancy in the data by compressing it to a lower dimension [54]. Koutroli *et al.* benchmarked this method against the KEGG database to evaluate the predicted interactions and showed a higher rate of true positive predictions compared to PCC-based methods. They also identified about 2000 interactions for 487 understudied proteins (the 10 % least published proteins). STRING DB uses the FAVA model to generate the co-expression interactions and report the confidence score.

Though the high-through nature of expression studies helps overcome the bias in experimental studies, it is only a proxy for protein presence and abundance which can skew the inferred interactions [55]. Upadhya *et al.* [56] demonstrated that pathway deduction from expression study overemphasizes the easily quantified proteins. They analyzed replicate profiles of cell lines and found only a moderate correlation between mRNA and protein abundance. They showed that proteins with consistent abundance measurements across all replicates also have a higher correlation to mRNA levels. This implies that measurement reproducibility contributes to the variance between mRNA and protein abundances. Thus they concluded that strong correlations between mRNA and protein levels in certain pathways may not be due to biological reasons but to how reliably those proteins are measured [56]. This work shows the need to understand the measuring technique in order to interpret the data appropriately. Powers *et al.* [57] show that when studying gene associations under several phenotype conditions, the differences in expression levels of each sample are often explained by noise and sampling bias, rather than phenotypic signal. They call for the “bigger is better” approach that includes several large gene expression databases to increase the likelihood each gene has been sampled in the on and off state several times. This is a similar approach that most pathway prediction algorithms adopt to overcome the missingness in the input PPI network, and further necessitates a network representation that stores metadata for each interaction.

## 3 Network embedding methods transform PPI networks

Machine learning methods rely on a vector representation of the input network to perform prediction tasks. Thus, we need methods to convert heterogenous PPI networks to vector representations that ensure nodes with shared edges or neighbors in the network are close in the embedding space [58]. Network embedding methods have become popular because of their flexibility and consistent performance across networks from several domains. Particularly for PPI networks, embedding methods have two strengths: creating low-dimensional vectors for each node reduces the impact of sparsity in the network and creating a latent representation that aggregates heterogeneous data types into one vector.

Embedding methods transform the complex, high-dimensional structure of networks into low-dimensional, dense node vectors of a fixed size, which are less sparse than the adjacency matrix. Though embeddings move to a lower-dimension space, many features of the network, such as the structural equivalence and community structure, are still preserved; nodes that have similar roles in the network structure are close in the embedding space and nodes within the same cluster in the network are embedded closely. This helps in identifying proteins that serve the same function across different pathways and finding groups of proteins that interact within one pathway. Node2Vec [26], one of the most common embedding methods, generates a set of random walks within the network. The normalized edge weight is used to define the transition probability for traversing the edge between the two nodes in the random walk. A key innovation of this method is the use of a second-order random walk, where the probability of following an edge leading to an unexplored node is different from the probability of returning to the previously seen node. The transition probability is scaled differently for the edge that leads back to the previous node and for those that lead to other nodes. Thus the scaling parameter can be tuned towards staying in a local neighborhood or towards walking over long paths within the graph. These sequences of nodes are used as training for a one hidden-layer neural network that predicts the likelihood of the node’s neighborhood, and this probability vector becomes the embedding. Direct embedding methods assume that the input network is complete; however we know this is not the case for the human PPI network, and leads to lower confidence of predictions made with these embeddings. Also, Heimann *et al.* showed that different runs of direct embedding algorithms on the same input graph can lead to different embeddings depending on the selection of random walks [59].

Deep learning methods embedding methods can capture the non-linear structure of the PPI network more effectively than the relatively linear direct embedding approach. Variational Graph Autoencoder (VGAE) is a deep neural network framework to learn the latent representations of the nodes; it uses a similar structure to the VAE, with an encoder and a decoder. For a VGAE, the encoder is typically a GCN that uses the vectors defining the neighbors of a node and performs an aggregation function to define the given node [60]. The decoder then learns to generate data that resembles the original input based on the distribution learned by the encoder. Both the encoder and decoder use non-linear functions, and ultimately generate embeddings that capture not only the observed interactions, but also the underlying structure of the network and potential unobserved interactions. VGAE can also use node features in the input data; the initialization of the node vectors can be defined by protein features, such as protein sequence, structure, tissue-type, or functional annotation. Yang *et al.* created signed-VGAE (SVGAE) to use the protein sequence as node features, and trained a VGAE deep-neural model (two linear layers followed by a softmax) to perform prediction of interaction edges. They showed a 99.1 % accuracy using this method to predict interactions (benchmarked against known interactions from KEGG) [61]. The SVGAE method achieves an increased performance by only considering high-confidence edges, while ignoring the uncertain edges. Though this increases performance when evaluated with a set of known interactions, this training strategy may limit the ability to detect interactions between under-studied proteins (as the confidence is typically correlated to the amount of data). Furthermore, they use a signed adjacency matrix, where a negative one is used to denote unlikely interactions. However, truly negative pairs are difficult to define [62] so this may further introduce false negatives. MPI-VGAE is another VGAE-based method that creates an embedding of PPI networks across ten organisms. They incorporate molecular features of the proteins in the initialization of the node vectors, and show that this method leads to the reconstruction of hundreds of metabolic pathways [63].

As mentioned in Section 2, independent data sources are commonly aggregated to address the issue of sparsity in the PPI network, and embedding methods are able to learn one singular vector to represent all the interaction data sources [61], [64], [65]. BIONIC [66] is the leading PPI network embedding method specifically designed for pathway prediction tasks. This method uses protein-protein interaction networks, genetic interactions, and networks derived from co-expression studies as input to layers of graph convolution to produce node vectors of length 512. A separate GAT is trained for each interaction type. For each interaction type, several layers of convolution are stacked together, and with each layer, the neighborhood size that defines a given node grows. This generates features that encompass higher-order neighborhoods. The output of each layer is summed to create the final vector. Forester *et al.* performed a hyperparameter optimization and found that three layers (summing the results when using neighborhood sizes of one, two, and three) is optimal. The interaction specific node features are then combined through a weighted summation. A scaling coefficient is learned for each interaction type and is applied to the node features before summation. A key feature of this combination process is the introduction of a mask, where node features from the interaction specific vectors are dropped randomly before the summation. This forces the encoders to compensate for missing node features, creating an integrated embedding that is robust to noise and missingness. The decoder then performs a dot product operation on the integrated embedding and attempts to reconstruct the networks (with all the interaction types). This process iterates to minimize the loss between the input network and the reconstructed network. Forster *et al.* [66] demonstrated that their embedding method produced novel interactions in yeast that could be experimentally verified, showing the utility of integrating several interaction sources into one vector representation for downstream analysis.

When considering the pathways that are known to lead to disease, they are often disconnected components in the PPI network, and thus the spatial clustering of these proteins in the embedding space is insignificant [67]. Agarwal *et al.* showed that the assumptions of the leading embedding methods, including GCN and random walk based methods, do not fully capture the network structure to bring these disconnected components together. However, they did conclude that the proteins in disease pathways do form high-order structures, such as cycles ranging from sizes three to six. They found that certain higher-order structures are more correlated with certain disease categories. Therefore, we conclude that more work in embedding subgraphs should be conducted, rather than focusing only on node-level embeddings. .

**Table 2:** Network Embedding converts complex, high-dimensional networks into low-dimensional node vectors, preserving network features and are the first step in pathway prediction algorithms that use heterogenous networks. These methods are used to reduce the impact of sparsity and aggregates heterogeneous data into one vector. Embedding methods assume a complete input network, which is not usually found with biological networks. Therefore, the type of embedding method chosen has impact on how the missingness in the network is addressed and the impact on the downstream predictions. This table details the common embedding methods and their strengths and limitations.

	Description	Input Data Description	Pros	Cons
Node2Vec	Generates random walks within the network to create node embeddings.	Network structure; normalized edge weight defines transition probabilities for random walks.	Preserves structural equivalence and community structure; tunable parameters for local/global walks.	Assumes complete input network; different runs can produce different embeddings; may lower prediction confidence.
Variational Graph Autoencoder (VGAE)	Learns latent representations of nodes using a GCN-based encoder and decoder structure.	Network structure; node features (e.g., protein sequence, structure, tissue-type, functional annotation).	Captures non-linear structure; uses node features; learns from observed and unobserved interactions.	Training may limit detection of interactions between understudied proteins; signed adjacency matrix can introduce false negatives.
MPI-VGAE	Embeds PPI networks across multiple organisms with molecular features initialization.	PPI networks of multiple organisms; molecular features of proteins.	Reconstructs metabolic pathways; utilizes cross-organism data.	Complexity increases with multiple organisms; requires comprehensive molecular features.
BIONIC	Embeds PPI networks using genetic interactions and co-expression studies, incorporating graph convolution.	Protein-protein interaction networks, genetic interactions, co-expression data.	Robust to noise and missing data; integrates multiple data sources; optimized for pathway prediction tasks.	High complexity; requires hyperparameter optimization; potentially high computational cost.

## 4 Advances and challenges in pathway prediction

Protein pathway prediction involves a series of subtasks, and the outputs collectively form a pathway. These tasks fall under two primary categories: (1) set prediction and (2) edge prediction. Set prediction identifies proteins from the entire proteome linked with a specific phenotype. Edge prediction finds the interactions within that set that create the phenotype. Each interaction represents a potential mechanistic link where one protein might activate, inhibit, or modify another, thereby driving the progression of a biological process. By accurately predicting these interactions, we can map out the sequence of events within the pathway, revealing the underlying regulatory mechanisms. In network theory, while domain-agnostic prediction models effectively perform set and edge predictions, they operate under the assumption that the input graph is balanced (similar degree across all nodes) and complete (all edges are present) [68], [69]. When applied to proteomic networks, with an estimated 80 % of edge missingness [21], the performance of generalized methods can be poor. In this section, we review prediction methods that overcome the challenges inherent to the PPI network topology.

### 4.1 Set detection algorithms

#### 4.1.1 Unsupervised set detection methods

Clustering methods categorize proteins into groups based on similar functions, hypothesizing that proteins with high interconnectivity in the interaction network, indicated by shared edges, neighbors, and paths, are likely to share functions. Embedding vectors are generated for each node such that nodes with common edges, neighbors, features, etc. are pushed together in the embedded vector space and nodes with no path between them are forced apart. The cluster is then defined using a distance threshold. This distance threshold is chosen to minimize the distance between objects within each cluster, ensuring that members of the same cluster are as close as possible, thereby enhancing intra-cluster similarity. Simultaneously, it aims to maximize the distance between different clusters, ensuring that each cluster is distinct and well-separated from others. This dual optimization process involves iteratively adjusting the cluster assignments and sometimes the cluster centers until a certain convergence criterion is met, effectively balancing the compactness of clusters with their separation from one another. The quality of the cluster is calculated using the path distance between proteins, the weight of the edges, and the data source that defines the interaction edges. Novel clustering methods build upon this standard approach to include variable interactions that lead to dynamic protein functional sets [70], [71].

Clustering is an effective approach for subgraphs of the interaction network with a consistent density of edges across all nodes. In consistently dense regions, there is approximately uniform information for each node, which allows the embedding algorithm to accurately encode the nuances of the network. For example, regions of the genome involving tyrosine metabolism, fatty acid degradation, and the p53 signaling pathway are highly studied and have an equal distribution of edges [72], and so clustering algorithms create discretized functional groups. Ke *et al.* used gene expression data, obtained from 10528 tumor and normal samples, to create protein embeddings and then partitioned the dataset into distinct, non-overlapping clusters. To create these clusters, they initialize random centroids, assign each data point to the nearest centroid, and update the centroids by calculating the mean of all points assigned to the cluster. This continues iteratively until the centroids no longer change. The studied proteins are implicated in cancer phenotypes, and the clustering method accurately and precisely distinguishes pathways that contribute to specific cancer types [73], [74]. Incorporating spatial transcriptomics data into the interaction network further increases the specificity of the pathway set prediction; Long *et al.* introduce GraphST to integrate spatial data as edge weights (such that spatially adjacent proteins have a shorter edge distance) and produce clusters that are delineated by fine-grained tissue structures in brain and embryo tissue [75]. Furthermore, Savage *et al.* showed that areas of the network that have the same number of data sources describing each protein allows the clustering method to overcome the influence of hub-nodes. They use multiple functional annotation sources, such as GO and KEGG, to create edges between proteins indicated in cancer pathways. They used a node affinity method, using the euclidean distance between embedding vectors to define centroids. At each iteration of the node affinity algorithm, each node’s vector is updated by a factor of its neighboring nodes’ vectors. Because of the consistent number of data sources per protein, they were able to create well-defined boundaries that correspond to known cancer pathways [76].

In areas of the network with sparse edges, clustering methods have decreased predictive power. Skinnider *et al.* [77] studied the impact of “hub-proteins” on novel functional group predictions by evaluating 17 PPI databases. They demonstrate a strong correlation between the node degree and the number of functional groups predicted. Skinnider *et al*. also observe that the experimental confirmation of predictions for these well-studied proteins is challenging. [77]. Haynes *et al.* [78] describe this phenomenon as the “rich getting richer”; they studied 104 human disease-related genes by clustering transcriptomic data to find that most of the proteins associated with a disease were novel and not contained within GO annotations [78]. They conclude that the literature focuses on well-annotated genes instead of those with the most significant disease relationship in terms of both expression and genetic variation. Therefore, for understudied proteins and functions, the imbalance of edges and lack of experimental annotation significantly impact the ability of clustering methods to make novel predictions [79]. As the availability of high-throughput data that measures the entire proteome increases, this annotation imbalance will decrease, and so this issue can be addressed by developing methods that can incorporate several data sources.

#### 4.1.2 Supervised set detection methods

Supervised machine learning methods, instead of depending on dense edge connections, train on high-quality, hand-curated pathways to predict novel sets. These algorithms identify essential characteristics from these examples and generate predictions encompassing these features. Thus, sets of functional nodes that do not have consistent degrees, such as star topologies and combinations of linear and clique topologies, can be recognized through the similarity of features to known pathway sets [80]. Supervised set detection methods are less influenced by the presence of hub-nodes, because the algorithm is optimizing for similarity to known pathways rather than just topological connectivity [80]. Supervised methods can achieve high accuracy and precision in predicting protein complexes by learning from labeled examples of known complexes, making them reliable for identifying specific types of complexes within PPI networks [81].

Supervised methods can integrate diverse protein data types (topological, expression, functional, sequence-based) and use the labeled examples to achieve high prediction accuracy. To integrate the datasource, these methods follow the typical embedding step to transform the input network into a low-dimensional representation; the type of supervised model used dictates the appropriate embedding strategy. Random forest prediction algorithms use embedding methods that directly capture the network properties. NodeEmbed-SLPC-RF [82] is one example of a supervised method that generates an embedding by simulating random walks through the graph to capture the local neighborhood as input to a random forest model. ClusterSS [83] uses a set of vectors that correspond to different network properties (e.g., sub-graph size, edge weights, clustering coefficients.), and these vector inputs for known pathway sets are used to train a neural network model. The neural network generates a score that is adjusted by a local structure score function at each iteration of the set search during inference; the neural network uses non-linearity to represent the latent features of the training examples which is combined with the a quantification of the network structure to increase the accuracy of the predictions.

Deep learning approaches are the most flexible in representing different data types, incorporating protein node annotations, typically GO annotations, into the embedding [84], [85]. These approaches use a message-passing approach for embedding the nodes, and the size of the models increases the generalizability of the model. For example, Super.Complex [86] is a deep learning method that implements an Automatic Machine Learning (AutoML) algorithm to perform set prediction. AutoML streamlines the application of machine learning by automating the selection of optimal algorithms and fine-tuning their hyperparameters. AutoML tools employ sophisticated search strategies, like grid search, random search, or more advanced methods such as Bayesian optimization, to explore the hyperparameter space efficiently. With this framework, Palukuri *et al.* searched through 6 different model architectures and hyperparameter spaces to choose the most predictive model for generating pathway set predictions in under annotated regions of the network. They created a set of synthetic networks to test the model; while using synthetic data is a common evaluation method, the calculated performance may not transfer to the desired regions of the protein network.

### 4.2 Edge prediction algorithms

Edge prediction specifically is important to the task of pathway prediction as the existing PPI networks are known to have 80 % missing edges [21]. Surprisingly, Kovacs *et al.* [87] demonstrated that the commonly held triad closure principle (that large numbers of common neighbors between two nodes implies a high probability that these nodes share an edge) does not hold for protein interaction networks because it overlooks the structural and evolutionary forces that govern PPI networks. Instead, pathway edge prediction methods need to include biological context, such as the role of interacting partners, post-translational modifications, cellular localizations, and chemical interactions, as well as topological structure in the node representations, to generate reasonable interaction predictions [88].

GNNs have advanced in recent years and are well-suited for link prediction (edge prediction) because they can integrate network structure features with node features and infer the relationship type between entities. GNNs harness non-linearities to uncover latent features within the data and make edge predictions in an end-to-end learning framework, that is advantageous in complex PPI networks [89]. Balogh *et al.* [90] implements a GNN that uses representations of induced subgraphs as training input to generate predicted edges. Because the training data is drawn directly from the PPI network and not specific to a particular domain, their method generates edges for all proteins across the proteome. It uses the sequence-based features and high-throughput expression features to find patterns between low-density edges and their high-density edge counterparts. The network learns to produce edge predictions that are indistinguishable from the distribution of known edges. Because the predictions appear to originate from the same distribution as known pathway examples, the method tends to exclude edge predictions in the sparsely annotated areas of the proteome [90], a limitation of supervised methods. BioNet attempts to overcome this limitation by using an encoder-decoder model [91]. The encoder uses several layers of graph convolution to learn the latent features of the training interaction. The decoder then uses these latent features to make link predictions between the proteins. The encoder and decoder iteratively update during the training process, which allows the link predictions to deviate from the known distribution.

An emerging area of interest is edge prediction methods specific to the spatiotemporal nature of the pathway [92]. Graph embedding methods have been extended to account for the change in network structure over time; Dynamic Graph Convolutional Networks (DGCNs) are extensions of GCNs that incorporate the temporal data as a feature for each edge, which enables the model to capture the evolution of edges over time. The DGCN convolves over the edges that are present at a given time step, and the updated edge and node vectors are used in the next time step’s convolution. These studies have been restricted to model organisms and bacteria, where the change in the interaction network is deduced from the change in expression pattern after an exposure to environmental factors. For example, Hedge *et al.* studied the network in bacteria after UV exposure and found several changes in topological structure that contribute to the cellular response [93]. With novel high-throughput sequencing technologies, human data with spatiotemporal annotation will allow for edge interaction predictions that are specific to particular cells and exposure. Magnano *et al.* conducted a preliminary case study in human fibroblasts to predict localized interactions involved in the pathway that responds to viral infection [94]. This type of dynamic data has been used in social networks to train a DGCN for link prediction, and these methods are applicable to the PPI network as well [95].

### 4.3 Emerging methods in sequential predictions

The sequence of proteins within a pathway denotes the cause-and-effect relationships of protein interactions, illustrating how one protein's activity can influence the function of another. Transformers, a type of deep learning model originally designed for natural language processing, can be leveraged to predict sequences of proteins that function together in biological pathways. By utilizing their ability to handle sequential data and capture long-range dependencies, transformers can model the complex interactions and regulatory mechanisms within protein networks. Graph transformers extend the transformer architecture to process graph-structured data, making them particularly well-suited for biological networks.

BERTwalk transforms graph structures into text-like sequences via random walks, and uses a transformer model to learn joint embeddings across different networks [96]. This method integrates three yeast and three human PPI networks that were generated with experimental and expression studies. The model then performs random walks through these networks to create an embedding, which is passed to a transformer model. BERTwalk's ability to predict pathway-level properties has outperformed previous methods like BIONIC in tasks such as gene co-annotation prediction and gene module detection on yeast and human benchmarks. Jha *et al.* first use the protein sequences to create node-feature vectors and the STRING PPI network as input to a BERT model to predict sequential interactions [97]. This model has demonstrated superior performance over existing approaches on multiple PPI datasets, highlighting the effectiveness of combining graph neural networks with amino acid-based features to predict sequences of interacting proteins.

The use of unsupervised learning is particularly notable in these transformer based methods, as they are adaptable to various biological datasets and capable of discovering latent patterns. The transformer based methods demonstrate significant improvements in predictingsequences of proteins and protein function within pathways. Therefore, generative models are useful in pathway predictions as they address the issue of cause and effect, also including understudied areas of the proteome. .

**Table 3:** This table provides a summary of methods used for edge prediction in protein-protein interaction (PPI) networks within heterogeneous graphs. Each row describes a distinct method, highlighting its description, the type of input data it utilizes, as well as its pros and cons. This comparative analysis aims to provide a comprehensive understanding of the strengths and limitations of each method.

Method Type	Description	Input Data Type	Advantages	Disadvantages
Unsupervised Clustering Methods	• Categorize proteins into groups based on similar functions • Using distance thresholds to define clusters	• Network structure • Embedding vectors	• Enhances intra-cluster similarity • Maximizes inter-cluster separation • Handles consistently dense subgraphs	• Decreased predictive power in sparse regions • Influenced by hub-nodes
Ke et al. [73]	• Uses individualized pathway activity measurement to classify functional groups (cancer subtypes)	• Gene expression data (tumor and normal samples) • KEGG pathway data	• Showed that individual gene experession levels can be used to precisely quantified the level of activity of each pathway in pan-cancer analysis	• Method has only been tested on well-studied regions, which high percent of pathway coverage
GraphST [75]	• A neural network based method with contrastive learning method that creates clusters delineated by fine-grained tissue structures	• Spatial transcriptomics data • Edges are weighted by the strength of correlation	• Can analyze multiple tissue stuctures concurrently • Fine-grained functional clusters	• High computational complexity when integrating spatial data • Requires high-quality spatial information
Savage et al. [76]	• Uses multiple functional annotation sources to create edges and employs a node affinity method with Euclidean distance for clustering.	• Functional annotation sources (GO, KEGG)	• Overcomes hub-node influence	• Requires consistent number of data sources per protein • Complex iterative process
Supervised Set Detection Methods	• Train on high-quality, hand-curated pathways to predict novel sets • Optimizes for similarity to known pathways rather than topological connectivity.	• Hand-curated pathways as training labels • Diverse protein data organized into a network	• High accuracy and precision • Less influenced by node degrees (hub nodes) • Integrates diverse data types	• Requires high-quality labeled data • Propogates experimental bias to predictions • Computationally intensive
NodeEmbed-SLPC-RF [82]	• Generates node embeddings using node2vec • Interaction edges are weighted by the similarity of embedding vectors • Labeled local neighborhoods are used as training data for random forest model	• PPI network • Labeled positive protein pathways • Labeled examples of protein sets that are not a pathway	• High performance in predicting functional protein sets from human and yeast PPI networks • Random Forests are a relatively simple, low-complexity model	• High dependence on network edges • Performance in understudied areas is not as high
ClusterSS [83]	• Extracts vector network features from true pathways • Extracted features are used to train a neural network for prediction • Adjust the neural network predictions and confidence with local structure information	• PPI network and local network structure properties • Labeled protein pathway examples	• Increased accuracy of pathway predictions • Evidence of prediction for novel functional groups (proteins not previously included in pathways) • Combines non-linearity with quantified network structure features	• Complexity of training of the neural network • Model is highly dependent on the type of embedding method used to create the feature vectors
Super.Complex [86]	• Implements an Automatic Machine Learning (AutoML) algorithm to perform set prediction, optimizing model selection and hyperparameters.	• Weighted PPI network • GO annotations • Protein node sequence annotations	• Highly parallelized implementation of 14 different models to find the optimal method • Generalizable to include new types of protein data	• Performance evaluated on synthetic data (may not transfer) • Limited discussion on performance on understudied areas of the proteome
Edge Prediction Algorithms	• Include biological context and topological structure to generate interaction predictions • Overcome missing edges in existing PPI networks	• Topological structure of biological networks • Node features, • Biological context	• Integrates complex network features • Suitable for predicting interactions in PPI networks	• Limited by availability of high-quality training data • Potential high false positive rates
GNN-based Edge Prediction [89]	• Uses representations of induced subgraphs as training input • Integrating sequence-based and high-throughput expression features to predict edges	• Sequence-based features • High-throughput expression features	• Generates edges across the proteome, learns patterns between low-density and high-density edges	• Excludes edge predictions in sparsely annotated areas • Requires large datasets
BioNet [91]	• Uses an encoder-decoder model • Encoder uses graph convolution layers to learn latent features of interactions • Decoder uses these features to predict links between proteins	• Heterogeneous biological network (can include node entities besides proteins)	• Captures latent features • Allows deviation from known distributions • Iterative updating improves prediction accuracy	• Very large model increases the computational complexity for training (requires substantial GPU resources to train)
Dynamic Graph Convolutional Networks (DGCNs) [93]	• Extend GCNs to incorporate temporal data as edge features • Captures the evolution of edges over time	• PPI network • Temporal data to annotate the PPI edges	• Captures temporal changes in network structure • Suitable for dynamic interaction predictions	• Restricted to model organisms and bacteria • Limited human data with spatiotemporal annotation
Graph Transformers	• Extend transformer architecture to process graph-structured data • • Suitable for biological networks.	• Graph-structured biological network data	• Handle complex network structures • capture long-range dependencies; suitable for biological pathways.	• High computational resource requirements; complexity in model implementation.
BERTwalk [96]	• Combines BERT masked language model with network propagation for graph representation learning by transforming graph structures into text-like sequences via random walks	• Heterogeneous biological network • Includes several different node types • Network propagation data	• Integrates various biological networks • Outperforms previous methods in gene co-annotation and module detection tasks	• Requires extensive computational resources • Large model size makes explainability challanging; hard to justify novel prediction
Graph-BERT [97]	• Feature vectors for each protein • Feature vectors for protein interaction pairs are concatenated • BERT model to encode the PPI network • Fully connected layer to classify interactions	• Features extracted from protein information • Protein sequences • PPI network structure	• Superior performance in multiple PPI datasets • Combines graph neural networks with sequence-based features. • Trained model can be used through open platforms	• Complexity in training and implementation • Limited to predicting sequences of pairwise interactions

### 4.4 Challenges with prediction and phenotype association

Deep learning and GNN prediction methods show promise in learning the latent features of known pathway sets; however, they are limited by the availability of high-quality training data. Larger models need an order of magnitude (100 K) more training examples than are currently stored in pathway databases (10 K). Moreover, high false positive rates and limited explainability make the prediction methods unreliable [98]. The field has not settled on standard benchmark methods; each study defines its own subset of the data to compare itself to other models’ performance. While this may create a self-consistent evaluation, it prevents true comparison of the outputs and performance of different models.

Predicting the phenotype associated with a set of nodes and edges remains an open-problem and is relatively unaddressed through network methods. Historically, this task has been accomplished using enrichment studies, where gene sets within a pathway are tested for significant association with a phenotype [99], [100]. Statistical epistatic networks are one step towards a network approach for pathway-phenotype predictions. In this type of network, each node corresponds to a genetic variant or SNP, and the edge corresponds to the interaction between the two SNPs [101], [102]. The edge annotation relies on enrichment studies to indicate the prevalence of an SNP interaction in a given phenotype. Large connected components in this network suggest a group of SNPs that together cause the disease outcome, as shown in bladder cancer and multiple sclerosis studies [101], [102]. However, most of these pathway phenotype prediction studies are tested using cancer datasets, which are richly annotated with control and case samples [103]. There is limited evidence that such methods are successful for more sparsely sequenced phenotypes [78].

## 5 Statistical methods and biological interpretations are needed to filter false positives from the predicted pathways

When using computational prediction methods, distinguishing significant potential discoveries from false positives is a challenge, and so statistical validations are performed on the final predictions. Significance evaluations are used to ensure the validity of the specific prediction model and that the rate of prediction is reasonable with respect to the degree of known interaction data for the proteins. Particularly when optimizing for de novo predictions, where limited reference data exists for direct comparison, biological evaluations, and relevance are crucial. This section will review machine learning and biological approaches to validate the results of computational pathway predictions.

### 5.1 Predictive power of the method

Machine learning methods typically rely on held-out, gold-standard validation sets that are used to determine the performance of the method. Creating a truly isolated validation set completely separate from the training data is a challenge in network-based methods [104]. When performing set prediction, the protein nodes that are included in the validation dataset should not be included in the training dataset. This limits the amount of training data available because proteins can participate in several pathways. Using an external source of data for validation is a common method to overcome this limitation. For example, Dilmaghani *et al.* use a database of gold-standard protein sets as the benchmark; however, they did not address the possibility of data leakage across the definition of the gold-standard sets and the features used for training the algorithm [105]. In the case of interaction (edge) prediction, known interactions can be removed from the training set, but this changes the underlying network structure, biasing the evaluation [106].

To measure the success of the validation set, learning methods typically report an Area under the receiver operating curve (AUROC) value, but because of the unevenly distributed edges in PPI networks, this metric will overestimate the performance [104]. Networks have a large number of negative examples (unconnected node pairs) in camoparison to the number of positive examples, and so a classifier that simply predicts no edge for every node pair would achieve a high AUROC. Instead, the Area Under the Precision-Recall Curve (AUPRC) is a more appropriate metric to use for evaluation. These evaluations address the difficulty of obtaining reliable true negatives; given the largely understudied proteome, defining true negative protein interactions and pathways is difficult. As a result, many methods that propose pathways evaluate their results on datasets that include both false positives and false negatives, which introduces statistical error [107]. Methods that use multiple data sources may improve these statistical assessments. Nguyen *et al.* created DANUBE [108] to combine statistics from individual studies to filter the proposed pathways, identifying a more specific subset of relevant pathways. Alam *et al.* [109] demonstrate the utility of multivariate analysis to validate pathway predictions from knowledge graphs built with several different features.

### 5.2 Measuring the relevance of the predictions

Though the biological relevance of the predicted pathways is most convincingly confirmed with experimental validation, computational approaches often rely on an a priori gene list to calculate a p-value, measuring the strength of association between a gene list and a phenotype [107]. Loopez-Ibanez *et al.* evaluated the significance of chemical structures in a biological pathway; they calculated the enrichment of a defined set of fragments within a pathway and used this information to score novel compounds given the presence of pathway-enriched fragments [110].

However, network-based predictions often begin without an a priori list of elements. Instead, node-level statistics, such as centrality, similarity measures, and probabilistic graph measures, are used to evaluate the likelihood of the pathway. These metrics are often combined, often with nonlinear functions, into a pathway-level score [111]. The pathway scores can then be used to assess the significance of the predicted pathway by comparing the score to the distribution of known pathway scores [112]. In network studies, however, it is challenging to build a null model for comparison, because randomizing the nodes or edges can influence the p-value calculations. Common random networks, such as Erdos-Renyi networks [113], do not follow the same distribution of node degrees observed in PPI networks [114], and thus are not a fully representative null model.

### 5.3 Biological experiments for confirmation

Biological experiments are the gold standard for verifying pathways. They are commonly performed with knock-out or knock-down studies. Protein knockout/knockdown studies use specific cellular engineering to remove a particular protein through accelerated proteolysis, RNA interference, antisense oligodeoxynucleotides, or ribozymes [115]. However, these methods can be limited because the procedures are designed to study individual protein characteristics. Biological validation is an expensive, laborious process that often results in inconclusive phenotypic observations at a system level [115].

Set detection predictions are node-centric predictions, and so the relevant experiments evaluate the protein function. When predicting that a new protein is involved in a known pathway, techniques such as co-immunoprecipitation can be used to demonstrate physical interactions with known pathway members, while gene knockdown or overexpression studies can elucidate the functional impact of this protein within the pathway [116]. For hypotheses suggesting a new set of proteins constitutes a new pathway, systematic approaches such as mass spectrometry-based proteomics can identify and confirm the involvement of these proteins in specific cellular responses or processes [117]. Edge predictions are confirmed by investigating interactions between two proteins within a pathway. These experiments typically use yeast two-hybrid assays or fluorescence resonance energy transfer (FRET) to demonstrate direct physical interactions [3]. Lastly, to link a pathway to a specific phenotype, genetic manipulation techniques such as CRISPR-Cas9-mediated gene editing can be employed to modify pathway components and observe resultant phenotypic changes in model organisms [118].

Interpretation of the prediction models that relate the latent features of the network to known biology can provide a proxy for biological validation [119], [120], [121]. Understanding the key features for a prediction model enables comparison with known biologically significant features; higher confidence is given to a model that places importance on biologically relevant features (e.g. domain regions of the protein, strong evidence for physical interaction between proteins). DeepClassPathway uses saliency maps to explain the features of importance to the deep neural network model trained to predict the pathway causing a patient’s specific Human papillomavirus (HPV) status. Lombardo *et al.* showed that the important protein features vary across HPV-positive and negative patients, and the most significant protein regions for the HPV-positive predicted patients correspond to known upregulated proteins for the depression pattern [122]. Innovations in the explainability of the prediction model show promise that the network is learning features that are biologically relevant to the pathway.

## 6 Opportunities in pathway discovery lie in combining data sources and emerging community detection methods that can account for heterogeneous data

Fundamentally, pathways consist of protein-protein interactions that are ordered sequentially to create a phenotype. We see two major opportunities in (1) incorporating unbiased multi-omics data to generate more specific PPI predictions and (2) using novel algorithms to generate de novo predictions.

High-throughput experimental technologies produce unbiased data for network analysis at scale. Shown by the innovations in the studies reviewed, this data is key to understanding the proteome and the relationship between expression and phenotype. Network methods provide a flexible framework that can represent heterogeneous data types, integrating different experimental results for each protein. Incorporating high-throughput expression studies, sequence and structure representations, and tissue and cell-type specificity with known interactions provides a holistic understanding of the protein while overcoming the bias present in any one data type. Using this heterogenous data to create subgraph embeddings, rather than just node-level embeddings, can better represent known pathway features. These representations can better capture the higher-order structure that defines a pathway which is useful when predicting novel pathways.

The bias towards well-annotated proteins is seen in both the prediction and evaluation of protein pathways. In prediction, the known pathways and highly studied proteins dominate the training data and thus may bias the predictions. The predictive ability of neural network algorithms is limited by the available training data and so current conventional methods reinforce the bias towards well-studied proteins. Instead exploratory algorithms, such as reinforcement learning, will be useful for identifying novel pathways in areas of the proteome, where example-based algorithms have limited classification power. Supervised methods are useful for training when the output is known, but emerging generative methods, particularly transformers, have shown significant potential in generating protein pathway predictions by leveraging their capability to handle sequential data and capture long-range dependencies. By transforming protein interaction networks into sequences, models like BERTwalk and Graph-BERT leverage this advanced architecture to predict new interactions and understand the underlying biological mechanisms. Generative models can overcome the bias compared to traditional supervised methods, mostly due to the size of the model, and can generate sequential predictions that is analogous to the cause and effect relationship of interactions within a pathway.

Pathway prediction methods are typically evaluated with either simulated data or domain-specific, curated datasets. Both of these strategies present issues. Simulated data has clear ground-truth positive and negative examples, but often does not fully reflect the complexity of biology. Datasets with well-studied pathways are incomplete, and so true negatives are hard to define. Pathways with no annotation may follow different patterns than those that have been well-studied. Thus, a clear set of benchmarks should be identified; a consistent set of known pathways, from the publicly available databases, should be held out from all pathway training algorithms so that comparison across methods is consistent. The held-out set should include well-connected known pathways in the PPI network and proteins linked to the same phenotype but currently disconnected in the network. Thus, methods can be evaluated by how their performance changes over the two types of network structure.

## Author Agreement Statement

We the undersigned declare that this manuscript is original, has not been published before and is not currently being considered for publication elsewhere.

We confirm that the manuscript has been read and approved by all named authors and that there are no other persons who satisfied the criteria for authorship but are not listed. We further confirm that the order of authors listed in the manuscript has been approved by all of us.

We understand that the Corresponding Author is the sole contact for the Editorial process. He/she is responsible for communicating with the other authors about progress, submissions of revisions and final approval of proofs.

## CRediT authorship contribution statement

**Gowri Nayar:** Conceptualization, Investigation, Visualization. **Russ B. Altman:** Supervision, Writing – review & editing.
